# Advancing Autism Research From Mice to Marmosets: Behavioral Development of Offspring Following Prenatal Maternal Immune Activation

**DOI:** 10.3389/fpsyt.2021.705554

**Published:** 2021-08-06

**Authors:** Danielle Santana-Coelho, Donna Layne-Colon, Roslyn Valdespino, Corinna C. Ross, Suzette D. Tardif, Jason C. O'Connor

**Affiliations:** ^1^Department of Pharmacology, University of Texas Health Science Center at San Antonio, San Antonio, TX, United States; ^2^Southwest National Primate Center, Texas Biomedical Research Institute, San Antonio, TX, United States; ^3^Audie L. Murphy Veterans Affairs, South Texas Veterans Health System, San Antonio, TX, United States

**Keywords:** maternal immune activation, translational model, vocalization, social behavior, non-human primate, behavior, prenatal, inflammation

## Abstract

Understanding the mechanism(s) by which maternal immune activation (MIA) during gestation may disrupt neurodevelopment and increase the susceptibility for disorders such as autism spectrum disorder (ASD) or schizophrenia is a critical step in the development of better treatments and preventive measures. A large body of literature has investigated the pathophysiology of MIA in rodents. However, a translatability gap plagues pre-clinical research of complex behavioral/developmental diseases and those diseases requiring clinical diagnosis, such as ASD. While ideal for their genetic flexibility, vast reagent toolkit, and practicality, rodent models often lack important elements of ethological validity. Hence, our study aimed to develop and characterize the prenatal MIA model in marmosets. Here, we adapted the well-characterized murine maternal immune activation model. Pregnant dams were administered 5 mg/kg poly-L-lysine stabilized polyinosinic-polycytidylic acid (Poly ICLC) subcutaneously three times during gestation (gestational day 63, 65, and 67). Dams were allowed to deliver naturally with no further experimental treatments. After parturition, offspring were screened for general health and vigor, and individual assessment of communication development and social behavior was measured during neonatal or adolescent periods. Similar to rodent models, offspring subjected to MIA exhibited a disruption in patterns of communication during early development. Assessment of social behavior in a marmoset-modified 3-chamber test at 3 and 9 months of age revealed alterations in social behavior that, in some instances, was sex-dependent. Together, our data indicate that marmosets are an excellent non-human primate model for investigating the neurodevelopmental and behavioral consequences of exposure to prenatal challenges, like MIA. Additional studies are necessary to more completely characterize the effect of prenatal inflammation on marmoset development and explore therapeutic intervention strategies that may be applicable in a clinical setting.

## Introduction

The etiology of neurodevelopmental disorders (ND) such as autism spectrum disorder (ASD) and schizophrenia remains poorly defined. Genetics play an important role in these disorders. Both heritable and *de novo* gene variations have been identified as causative factors in abnormal neurodevelopment (1, 2). Additionally, studies in monozygotic twins reveal a concordance rate between 60 and 91% for ASD (3), and 41 to 65% for schizophrenia (4). While dizygotic twins present a low concordance rate (5) demonstrating the considerable role of genetic in these disorders. Importantly, myriad environmental factors have been identified to play a putative role in the disruption of neurodevelopment. Prenatal exposure to drugs (6), stress (7, 8), environmental pollutants (9), and infection (10, 11) are linked to increased risk of ASD or schizophrenia.

During the last few decades, research indicates that stimulation of the maternal immune system during pregnancy may lead to, or increase the risk of, behavioral deficits after birth and into adulthood. Polyinosinic polycytidylic acid (poly IC) is a synthetic double-stranded RNA that, when administered experimentally, mimics a viral infection by activation of Toll-like receptor 3 (TLR3). In mice, maternal immune activation (MIA) with poly IC during early gestation (1st and 2nd trimester) causes behavioral deficits in offspring consistent with symptoms of NDs, such as repetitive/stereotyped behavior, deficits in sociability, communication, and sensory gating (12). Additionally, studies in rodents and primates have revealed cellular alterations in the MIA model similar to what is observed in humans affected by abnormal neurodevelopment, such as decreased parvalbumin-positive interneurons (13), altered synaptic pruning (14), and increased striatal dopamine (15).

Even though significant knowledge has been acquired through rodent studies, these models lack important elements of ethological validity and may have limited utility in understanding clinical pathology and developing therapeutic approaches. In 2006, a study carried out by Hackam and Redelmeier showed that just 36.8% of treatments that have been proven to be effective in rodent studies were replicated in human randomized trials (16). Considering this, along with the complex social and behavioral nature of ASDs, investigating the pathophysiology of the MIA-induced developmental disruption in a more translationally relevant model is necessary. An important feature of neurodevelopmental disorders is deficits in social behavior. Although several studies in rodents, including in maternal immune activation models, have identified molecular, cellular, and neural mechanisms involved in sociability, there are some critical differences between primates and rodents that make it unlikely to translate these findings. For instance, primate's social behavior depends mainly on visual and vocal communication while for rodents, other than auditory recognition, chemical and olfactory factors play a major role in social interactions.

The initial effort to develop the poly IC MIA model in non-human primates occurred in rhesus macaques. While these studies effectively recapitulated many elements of the rodent literature, they require navigating a number of practical challenges such as the prolonged developmental timelines where sexual maturity is not reached until 3–4 years of age, where young adulthood is not reached until after 5 years of age, and where housing requirements present limitations regarding the assessment of social behaviors (15, 17). Further, a comprehensive review of the literature by Amici et al. (18) suggested that most developmental changes in macaque sociality occur between 2 and 3 years, making logistics and cost of colony management for experiments particularly challenging. In order to overcome this challenge, we aimed to develop and characterize a translationally relevant and practical non-human primate model of maternal immune activation using marmosets.

*Callithrix jacchus* (common marmoset) are small New-World monkeys that have been increasingly utilized in neuroscience studies. This species presents several practical advantages when compared to other monkeys, such as their small size, relatively short gestation, and family group housing with a high degree of cooperative care and food sharing, which makes the investigation of complex social dynamic within the family group possible (19–22). Additionally, marmosets usually have multizygotic twin or triplet births with a unique chimeric placenta, which affords the opportunity to better understand how sex influences response to the same prenatal environment (23). Most importantly, marmosets are an excellent preclinical model to study immunopathogenic mechanisms in the prenatal and postnatal period as this species has a similar immune system to humans (24–26), and emerging research suggests that they may be suitable for genetic manipulation, such as targeted gene deletion/insertion, conditional gene expression, or mutation (19). Overall, marmosets are a unique model to study the effects of prenatal inflammation in dyzigotic or multizygotic twins. In humans the concordance rate of dizogotic twins for neurodevelopmental dizorders such as autism is low (5). There is a need of translational models that can be used to investigate the effects of environment in developmental programming. Hence, marmosets are a good candidate considering all the characteristics discussed above.

In the present study, we have performed an initial characterization of the MIA model in marmosets. Pregnant dams were administered poly ICLC subcutaneously on days 63, 65, and 67 of gestation, which corresponds approximately to the GD12.5 neurodevelopmental time point often used in mouse studies and to late first trimester in humans (26, 27). After Poly ICLC administration, plasma samples were collected to confirm the maternal immune response, and gestation was left undisturbed thereafter. After birth, general health, communication development, and social behavior were assessed in the offspring until adolescence to investigate if prenatal inflammation could lead to deficits in behavior. Our data confirm that MIA did not increase fetal/neonatal mortality rates or cause any overt deficits in reflex or motor coordination. However, similar to data from rodent studies and rhesus monkeys (12, 17), marmosets born from MIA dams exhibited subtle but significant disruption in vocalization (communication) development, whereby they exhibited significantly fewer cry calls when separated from their home cages as compared to non-MIA controls. Additionally, MIA offspring exhibited a distinct social behavior profile at both 3 and 9 months of age compared to controls. Together, these data are the first to establish the utility of the poly IC MIA model in marmosets.

## Methods and Materials

### Subjects

Common marmosets (*Callithrix jacchus*) used in the study were housed at the Southwest National Primate Research Center (SNPRC), Texas Biomedical Research Institute, San Antonio, TX. Animals were housed in a social family group with the temperature maintained between 76 and 84 degrees Fahrenheit and a 12 h light-dark cycle. Food was available *ad libitum*. The project was approved by the Institutional Animal Care and Use Committee at Texas Biomedical Research Institute (Approval number 1581CJ5). A total of 11 pregnant dams, with one dam contributing to both control and treatment pregnancy, and 19 infants were used in the study. All dams had at least one full-term pregnancy and reared infants before they were added to the study (Supplementary Table 1).

### Treatment

Pregnant marmosets were randomly assigned to either no treatment/0.9% sterile saline, or poly ICLC challenge. Gestational age was estimated by ultrasound with a possible error rate of +/– 3 days. An initial dose-response pilot experiment (0.32, 1, and 3.2 mg/kg of Poly ICLC s.c.) was performed using 3 non-pregnant adult female marmosets to establish a minimum effective immunostimulatory dose (data not shown). At gestational day 63, 65, and 67, pregnant dams received a subcutaneous injection of 5 mg/kg of polyinosinic-polycytidylic acid (Cat# P9582, Sigma Aldrich, St Louis, MO, USA) stabilized with poly-L-lysine (Poly ICLC) (28) which was prepared fresh for each 3-day injection regimen. A baseline blood sample was collected between gestational days 59 to 61 and 2 h after the treatment at gestational days 63 and 67. In order to boost the number of available pregnancies in this pilot study, four pregnant dams were enrolled immediately upon approval but were past the gestational time of saline injections. Three pregnant marmosets were treated with saline and 8 pregnant were treated with Poly ICLC. As the phenotype of infants from these unchallenged pregnancies was no different than those born to saline-injected controls, the two groups have been collapsed into a single control group throughout the study. Results were analyzed as % of baseline to normalize for inter-individual variability.

### Multiplex Cytokine Analysis of Maternal Plasma

The level of cytokines and chemokines on plasma samples collected from pregnant marmosets treated with saline or Poly ICLC were analyzed by Luminex assay using the protocol for New World primates established by Giavedoni et al. (29). The targets of the assay were Interferon-γ (IFN-γ), interleukin 17 (IL-17), tumor necrosis factor α (TNF-α), monocyte chemoattractant protein 1 (MCP-1), macrophage inflammatory protein 1-α (MIP-1α), macrophage inflammatory protein 1β (MIP-1β). Analytes are presented in the study when reliably quantifiable above the limit of detection.

### Neonatal Health and Vitality Evaluation

The Marmoset Assessment Tests (Matscore) were conducted 24–36 h after birth, as previously described (30). This behavior scoring method evaluates the newborn's motor and sensory skills. Briefly, infants were removed from their family group and placed in a surrogate (a small stuffed animal) until tests began. Removal of infants from the colony occurred by capturing the individual that was carrying the infant in the home cage and transporting both to the testing room inside a nest box. Following transportation, the infant was carefully separated from the carrier and the older animal was transported back to the colony while the infant was tested. The following behavior tasks were performed: crawling, clasping/righting 1, clasping/righting 2, vertical orienting, rooting, and auditory orienting. Full details of the behavioral tasks are outlined in Table 1. Offspring weight was recorded at the dates that behavioral assessment was performed.

**Table 1:** Matscore behavioral tasks description [Adapted from Tardif et al. (30)].

**Task**	**Score**	**Performance for a perfect score**
Crawling	0–1	1 if crawling within 2 min
Clasping/Righting 1	0–4	4 if grasping fur, maintaining ventral contact, and righting itself onto upper side of cylinder
Clasping/Righting 2	0–4	4 if grasping fur, staying in contact during quick movement and righting onto upper side of cylinder
Vertical orienting	0–2	2 if rotating 180° within 60 s
Rooting	0–1	1 if turning head and placing mouth on tip
Auditory orienting	0–1	1 if turning head in either direction

### Behavioral Characterization

#### Vocalization Recording and Analysis

**Figure 1:**
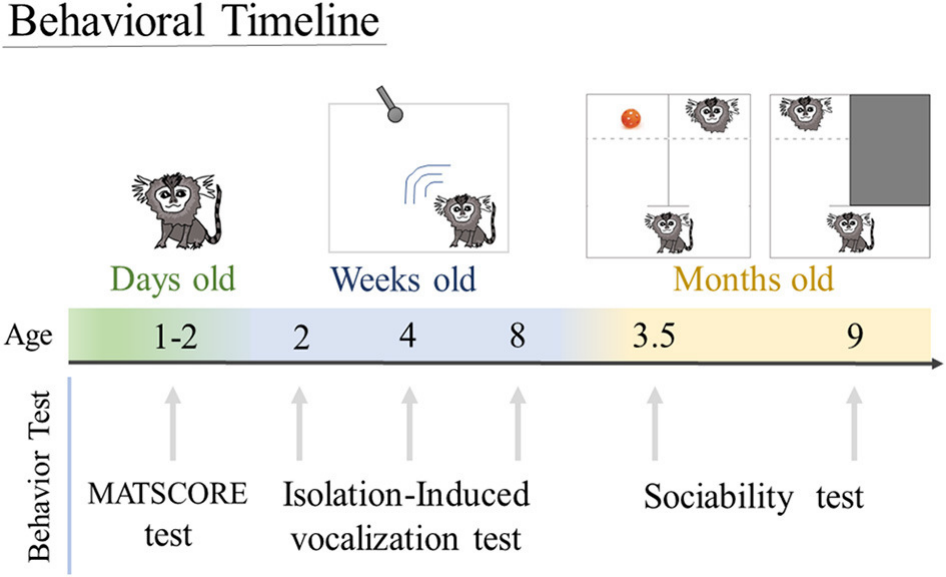
Behavioral timeline. Animals were tested to assess motor and sensory skill in the MATSCORE test when they were 1 to 2 days old. Isolation-induced vocalization test was performed when the infants were 2, 4, and 8 weeks of age. Sociability test was done at 3.5 and 9 months of age.

Offspring communication development was evaluated at the age of 2, 4, and 8 weeks old (Figure 1). On the test day, infants were separated from their social group and placed inside the recording chamber in an adjacent room. During vocal recordings, the animals had no visual contact with the colony but were likely able to hear the vocalizations originating in the adjacent colony room. After 10 min of recording, the infants were placed back in their home cage. Vocalizations were recorded using an Ultramic200K microphone and Seawave 2- Sound Emission Analyzer Wave edition (CIBRA and AEST, Italy) software using a sampling rate of 192 kHz. Manual spectrogram analysis was performed by a blinded rater using Audacity^®^ software 2.3.2 (31). Call types included in the present study were cry, trill, trillphee, twitter, and phee (**Figure 5A**). Calls were classified based on the classification by Jones et al. (32). Cry: call that appears as multiple harmonics, phee: monosyllabic call with a steady frequency, trill: monosyllabic call that is modulated going “up” and “down,” trillphee: call that starts as a trill and ends as a phee (32). To evaluate vocal development, the total number of calls, average call duration, and percentage of each type of call by age were calculated. All infants tested for vocalizations were also tested for social behavior.

#### Sociability Assay

An adaptation of the mouse three-chamber assay (33) was developed to evaluate social behavior in marmosets at 3.5 and 9 months of age (Figure 1). Subjects were habituated to the empty testing box for 20 min on two consecutive days. On the third day, after 10 min of habituation, marmosets were subjected to two 20 min tests: 1- social preference and 2- stranger interaction. In the social preference test, the infant was free to stay in the start area or enter one of two chambers within the testing box. In one chamber, the subject could interact through a wire partition with an older sibling from the same home cage, or in the other chamber, a familiar toy from the home cage was placed across the wire partition. In the stranger response test, the subject was returned to the start area while the sibling and toy were removed. Then an unfamiliar non-breeding adult was placed behind the partition of one chamber while the other chamber door was kept closed. The subject was then allowed to either stay in the start area or approach/interact with the stranger. We aimed to use same-sex siblings and strangers as the subject being tested. Whenever that was not possible, the sex of the stranger was matched to the sibling to avoid any sex preference that the tested subject might have. The location of the sibling/toy and stranger were randomly selected to control for potential side bias.

### Statistical Analysis

All data were analyzed using Graphpad Prism 8.3 (San Diego, CA). For time response curve data, Two-way ANOVA with repeated measures was used with age and treatment as the two factors. Two-way ANOVA with Bonferroni *post-hoc* was used to evaluate differences between sex responses, with sex and treatment as the two factors. One way-ANOVA was used to evaluate differences between the three groups (no treatment, saline-treated, and Poly ICLC). No statistical difference was detected between saline-treated or untreated dams for any outcome variable measured, so these groups were collapsed into a control group in order to enroll additional untreated pregnancies that were identified too late to administer the saline injections. This allowed a boost to the numbers in the control group within the time frame of the project period. Differences between the two groups were evaluated with Student's *T*-test when the distribution was parametric and Mann–Whitney test when the data distribution was non-parametric. Paired data non-normally distributed were analyzed with Wilcoxon matched-pairs signed-rank test. Normally distributed paired data were analyzed with a paired *T*-test. Parametric data were expressed as mean ± SEM, and non-parametric data were expressed as a median ± interquartile range. Data were considered significant if *p* < 0.05. Spurious data were identified in normally distributed data sets using Chauvenet's outlier test and omitted from analysis (34).

## Results

A dose-dependent immune response to Poly ICLC treatment was confirmed in non-breeding adult female marmosets in a pilot experiment performed prior to MIA of pregnant dams. 3.2 mg/kg was identified as the minimum effective dose to cause an increase in the levels of the inflammatory cytokines/chemokines TNF-a (261.5% ± 70 of baseline, *p* = 0.0278), MIP-1b (154.3% ± 69 of baseline, *p* = 0.0286), IFN-α (2,450% ± 2,250 of baseline, *p* = 0.0286), MCP-1 (228.1% ± 51 of baseline, *p* = 0.0321) from baseline. For experiments using pregnant marmosets, a slightly higher than the minimum effective dose of 5 mg/kg poly ICLC was used to induce MIA. TNF-α levels were measured to confirm MIA. Mixed-effects analysis revealed a significant main effect of poly ICLC treatment (*p* = 0.034) when comparing TNF-α levels between control or poly ICLC challenged dams. TNF-α levels were unchanged in the control group across time in response to vehicle injection when compared to baseline values. While, in the poly ICLC group, TNF-α levels were significantly increased 656.6 and 285% above baseline after the first or third poly ICLC injection, respectively (*p* < 0.05; Supplementary Figure 1). Although poly ICLC induced sickness behavior was not the focus of this study, it is notable that body temperature was not significantly changed by treatment. No observable effect on eating or home cage activity was apparent upon visual observation, although neither were systematically quantified. Analysis of parental age and weight showed that there were no significant differences between the control (no treatment), saline control, and poly ICLC groups. Further, litter size did not differ significantly between groups [*F*_(2,3)_ = 1.356, *p* = 0.2919 (Table 2)], and only two infants per litter were enrolled in the study as a surviving third must be placed with a foster dam per colony management guidelines.

**Table 2:** Summary of parental characteristics and pregnancy outcomes.

**Group**	**Dam's age (months)**	**Dam's weight (grams)**	**Sire's age (months)**	**Sire's weight (grams)**	**Litter size (*n*)**
No treatment control	49 ± 5.753	488 ± 40.87	111 ± 38.68	392.2 ±27.53	2.8 ± 0.20
Saline control	40 ± 4.583	577 ± 50.62	46.333 ± 6.119	430 ± 39.56	1.667 ± 0.881
Poly ICLC	42.75 ± 4.999	480.5 ± 24.18	72.5 ± 16.49	442.75 ± 23.66	1.875 ± 0.440

*Representation of the average of maternal age, maternal weight, paternal age, paternal weight, and litter size. Data is represented at mean ± SEM. Data were analyzed using One-way ANOVA with followed by Tukey post-hoc*.

**Figure 2:**
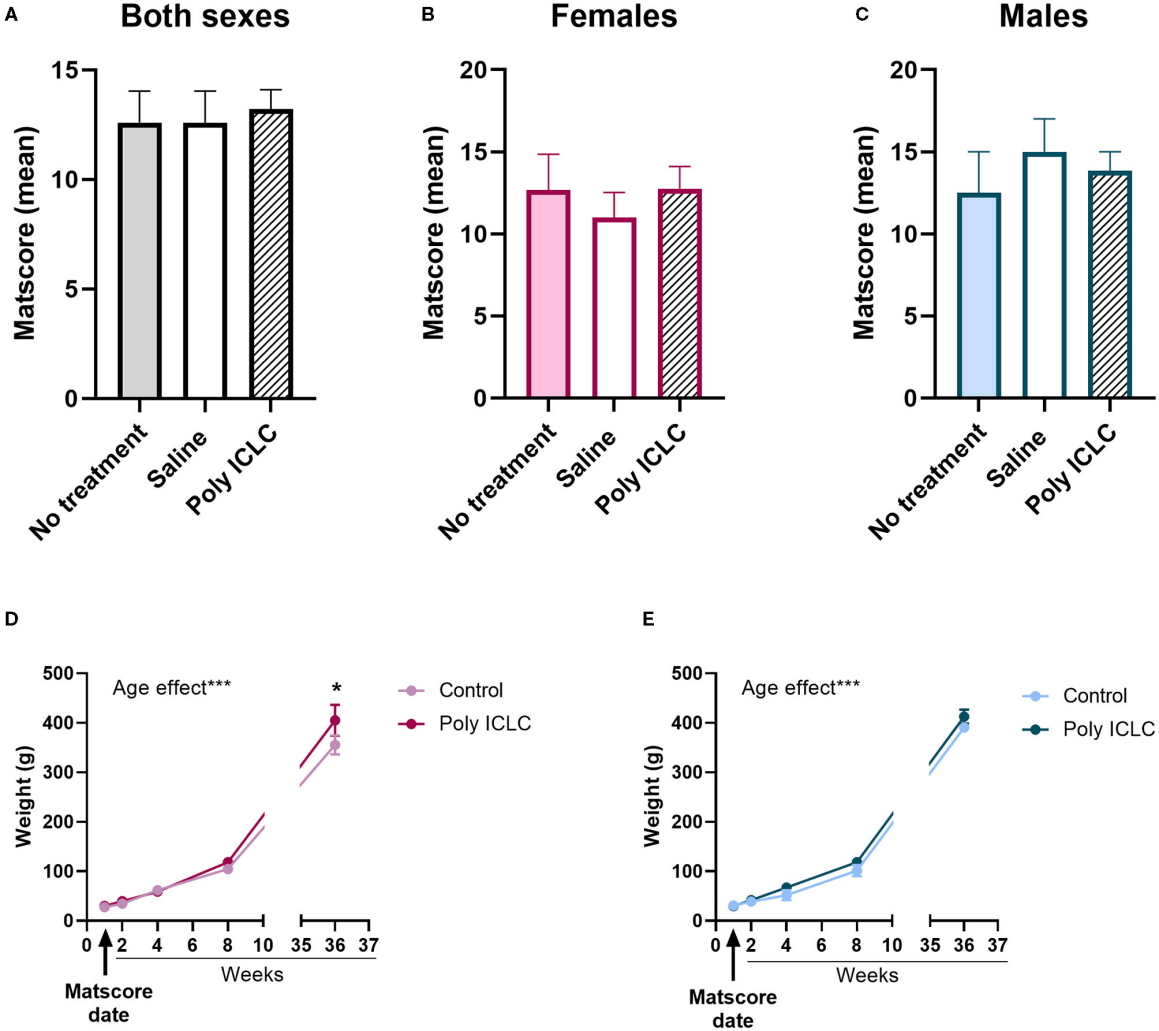
Offspring Developmental outcomes. Matscore test results at the first couple of days of age in **(A)** male and female offspring together, **(B)** female offspring alone, or **(C)** male offspring alone. **(A)** No treatment group *n* = 5, Saline group *n* = 5, Poly ICLC group *n* = 7. **(B)** No treatment group *n* = 3, saline group *n* = 3, Poly ICLC *n* = 4. **(C)** No treatment group *n* = 2, saline group *n* = 2, and Poly ICLC *n* = 3 Data are expressed as mean ± SEM analyzed with One-Way ANOVA. Offspring weight was recorded from MASTSCORE date to the 37th weeks of age in **(D)** female and **(E)** male offspring. Data are expressed as mean ± SEM analyzed with Two-Way ANOVA with repeated measures followed by a Bonferroni *post-hoc*. *p < 0.05 and ***p < 0.001.

Within the first 48–72 h after birth, the infant's general health, vitality, and neurodevelopmental rigor were assessed using the Marmoset Assessment Test (Matscore). Details of the test can be found in Table 1. Analysis of both sexes combined [Figure 2A, *F*_(2,11)_ = 0.7775, *p* =0.4833], just females [Figure 2B, *F*_(2,5)_ = 0.004, *p* = 0.9959] or only males [Figure 2C, *F*_(2,3)_ = 1.491, *p* = 0.3552] showed there were no statistical differences in the infant's MATSCORE performance between the different groups. Also, the performance on the different tasks of the MATSCORE was similar between the groups (Supplementary Table 2). According to a previous study with non-human primates, treatment with saline does not cause developmental deficits or immune response when compared to saline-treated monkeys (35). Additionally, there were no differences in the performance of the tasks in the MATSCORE test between saline and non-treated controls (Supplementary Table 2). Thus, offspring from these two groups were both included in a single control group. To continue evaluating the offspring's health development, we measured each offspring's weight prior to any experimental assessment of behavior. Male [Figure 2E, age effect, *F*_(1.811, 5.432)_ = 840.1, *p* < 0.0001] and female [Figure 2D, age effect, *F*_(4,37)_ = 298.7, *p* < 0.0001] offspring from all groups gained weight from the date that their MATSCORE test was performed until their 37th week of age. No differences in the weight gained were found between control and Poly ICLC groups in males [*F*_(1,3)_ = 4.200, *p* = 0.1328]. However, in females, poly ICLC offspring were heavier than controls at 37 weeks of age (*p* = 0.0310, Figure 2D).

**Figure 3:**
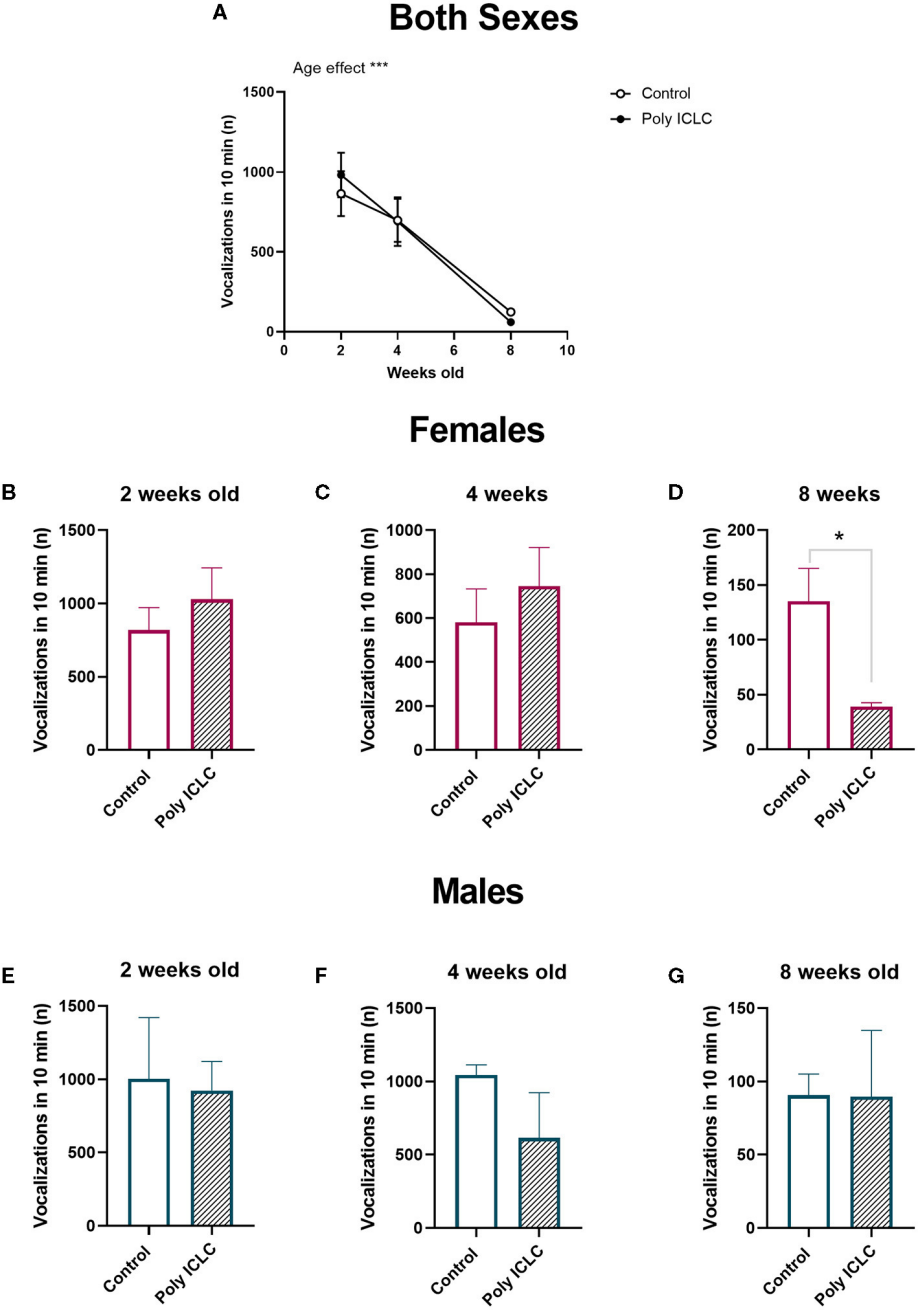
Total number of vocalizations decreases through development. **(A)** Total number of isolation-induced vocalizations performed by the infants at 2, 4, and 8 weeks old during 10-min recordings. Control *n* = 8 and Poly ICLC *n* = 7. Data are expressed as mean ± SEM and analyzed using Two-Way ANOVA repeated measures with Bonferroni *post-hoc*. Total number of vocalizations performed by female infants at 2 weeks old **(B)**, 4 weeks old **(C)**, and 8 weeks old **(D)**. Control *n* = 6 and Poly ICLC *n* = 4. Total number of vocalizations performed by male infants at 2 weeks old **(E)**, 4 weeks old **(F)**, 8 weeks old **(G)**. Control *n* = 2 and Poly ICLC *n* = 3. Data are expressed as mean ± SEM and analyzed using Students *T*-test. Significant differences are indicated by *p < 0.05 and ***p < 0.001.

Evaluation of vocal development was made by assessment of infants' performance in an isolation-induced vocalization test at 2, 4, and 8 weeks of age. During the test, infants were taken to an adjacent room and placed inside a sound-attenuating chamber, but they were likely able to still hear vocalizations originating at the colony while were not able to have direct visual contact. During the recording time, all marmosets performed vocalizations. Similar to previous reports in the literature (36), the total number of isolation-induced vocalizations emitted declines rapidly with increasing age [Figure 3A, age effect, *F*_(2,26)_ = 24.77, *p* < 0.0001]. The total number of vocalizations was not different between groups when both females and males were grouped together. However, when separated by sex, female marmosets (Figures 3B–D) from the poly ICLC group emitted fewer vocalizations than control females at 8 weeks of age (t_8_ = 2.544, *p* = 0.0345; Figure 3D). While for males, no significant differences were found at any of the ages we recorded their vocalizations (Figures 3E–G).

**Figure 4:**
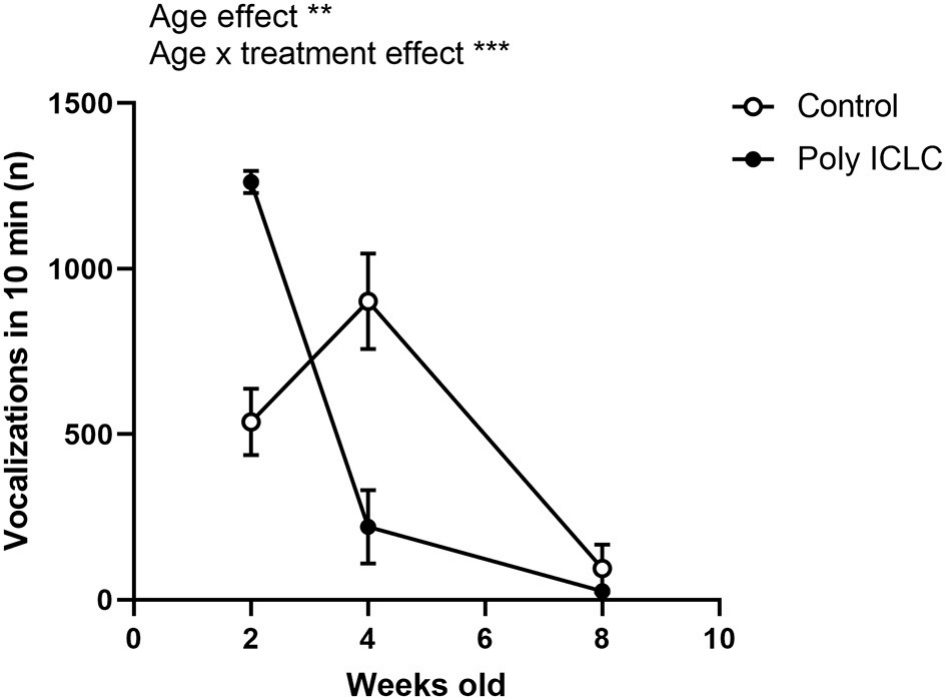
Comparison of the total number of vocalizations in different litters from the same dam. Total number of vocalizations performed by 2, 4, and 8 week old marmosets. The offpring are from one control litter and one poly ICLC litter that were born from consecutive pregnancies. Data are expressed as mean ± SEM and analyzed using Two-Way ANOVA repeated measures with Bonferroni *post-hoc*. Control *n* = 2 (females) and Poly IC *n* = 2 (1 female and 1 male) Significant differences are indicated by **p < 0.01 and ***p < 0.001.

One dam provided both a control litter and a poly ICLC litter to the study allowing a preliminary direct comparison between siblings to assess how important the genetic factor may be in the development of the phenotype. We analyzed the total number of vocalizations performed by different litters from the same dam, which revealed an effect of age in the total number of calls [Figure 3, *F*_(1.198,2.397)_ = 106.7, *p* = 0.0047]. Interestingly, there was also an interaction between age and treatment [Figure 4, *F*_(2,4)_ = 74.34, *p* = 0.007). At 2 weeks of age, the poly ICLC litter exhibit a higher number of vocalizations that decrease by 4 weeks of age, and further decrease by 8 weeks. While in the control litter, the number of vocalizations started lower than the treated litter, increased at 4 weeks, and then decreases at 8 weeks old (Figure 4).

**Figure 5:**
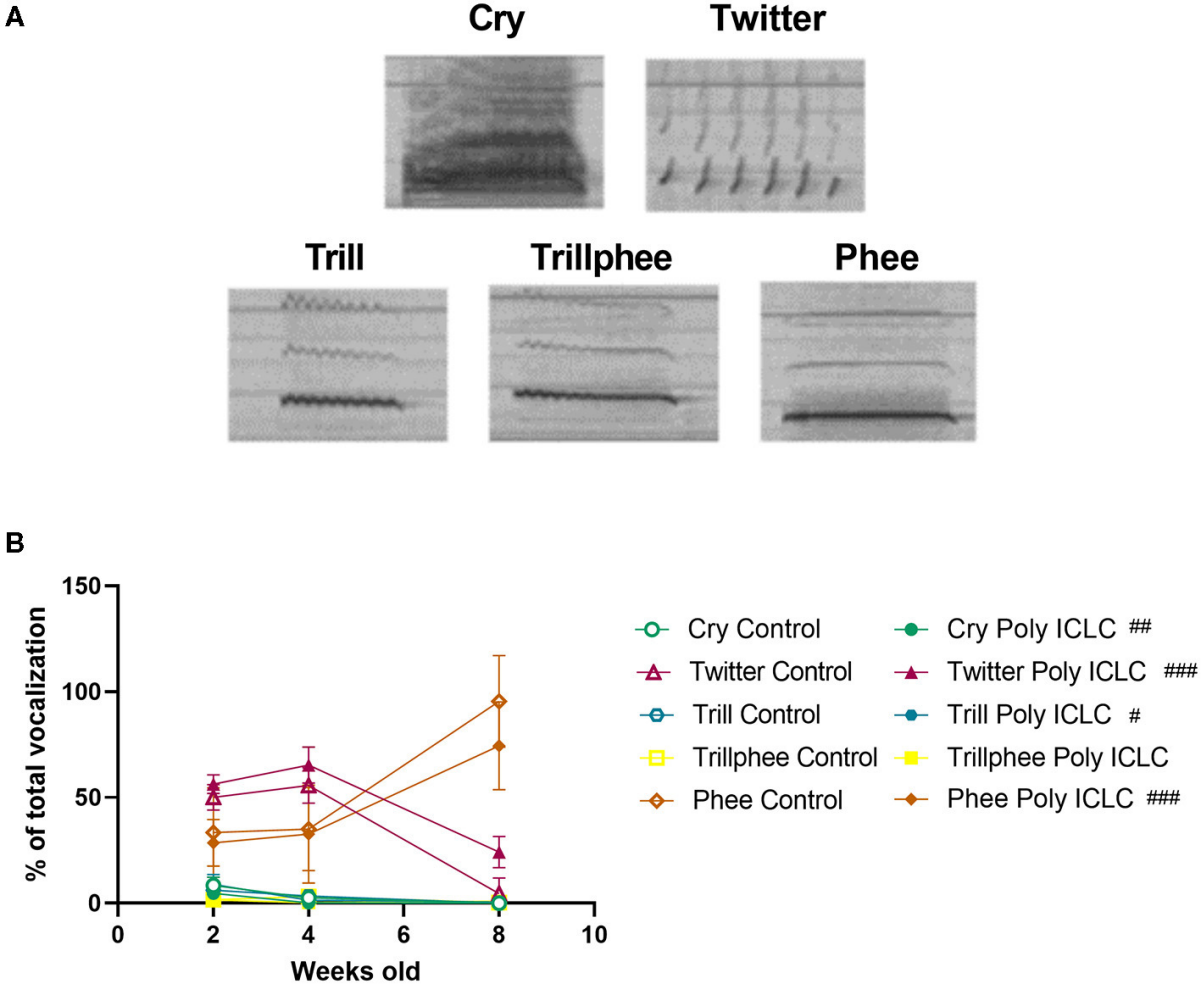
Infants' repertoire changes during development. **(A)** Spectrogram of the main calls identified in the vocalization recordings. **(B)** Proportion of the main calls performed by the offspring throughout development. Data was analyzed by Two-way ANOVA repeated measures with Bonferroni *post-hoc*. Control *n* = 8 (6 females and 2 males) and Poly ICLC *n* = 7 (4 females and 3 males). Data are expressed as mean ± SEM. Significant age effects are indicated by ##*p* < 0.01 and ###*p* < 0.001.

**Figure 6:**
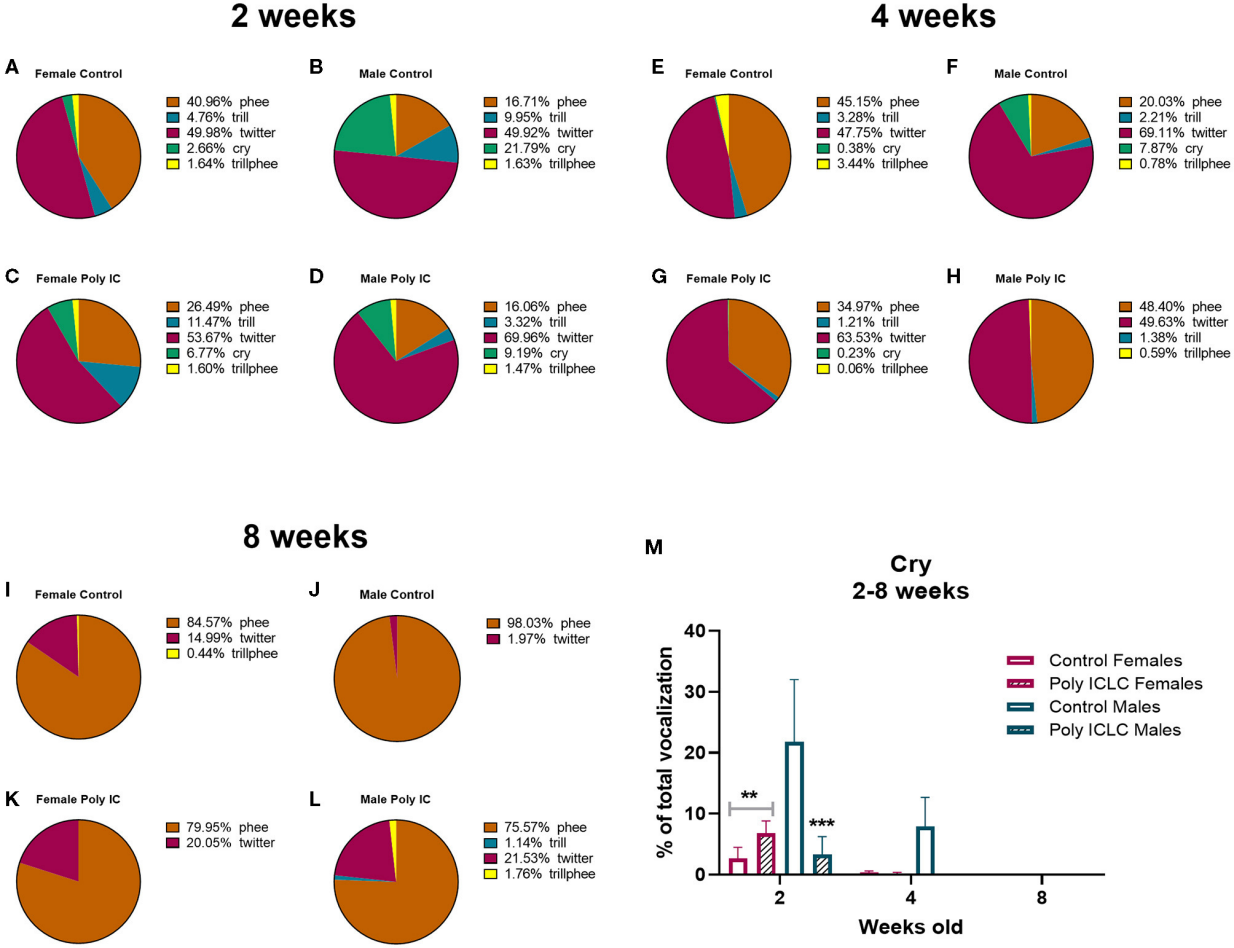
Infants' repertoire changes during development by sex. Repertoire composition in female and male offspring at 2 **(A–D)**, 4 **(E–H)**, and 8 weeks of age **(I–L)**. **(M)** Comparison between percentage of cry calls in female and male repertoires throughout development. Data expressed as average percentage of calls from the whole number of calls in the repertoire ± SEM. Data was analyzed by Three-way repeated measures ANOVA with Tukey *post-hoc*. Control female *n* = 6, Poly IC females *n* = 4, Control males *n* = 2, Poly IC males *n* = 3. Significant differences are indicated by **p < 0.01 and ***p < 0.001 vs. Control males.

In addition to the total number of calls, an important parameter that is used to evaluate vocal development is the vocalization repertoire. In our recordings, the predominant calls identified were cry, twitter, trill, trillphee, and phee (Figure 5A). From 2 to 8 weeks of age, most of the calls decreased in their prevalence, such as twitter that had an age effect [*F*_(2,26)_ = 18.41, *p* < 0.0001). Interestingly, phee was the only type of call that increased in prevalence with time [*F*_(1.905, 24.77)_ = 29.13, *p* < 0.0001; Figure 5B]. The distribution of the repertoire illustrated in pie charts suggests a dimorphic vocal distribution and MIA-effect in vocalization development (Figures 6A–L). Control males are the only group that still has a significant percentage of cry calls at 4 weeks of age when separated from the home cage, over 7% of their calls are cry calls (Figures 6E–H,M). Also, this group has a higher prevalence of phees (98.03%) at 8 weeks of age (Figures 6C,I–L). At 4 weeks of age, male MIA-offspring also has the least diverse repertoire presenting only four types of calls while the other groups present five (Figures 6E–H). However, at 8 weeks old, this same group has the highest diversity of calls when compared to the other groups (Figures 6I–L), suggesting that the male MIA infants present a delay in the development of their repertoire. A Three-way ANOVA showed an interaction of gender and treatment [*F*_(1,9)_ = 17.89, *p* = 0.0022] and age x gender x treatment [*F*_(2,9)_ = 7.713, *p* = 0.0112] when analyzing the development of cry vocalizations throughout time (Figure 6M). Interestingly, at 2 weeks of age, control males had the highest proportion of cries when compared to male poly ICLC (*p* = 0.0003) or females independent of treatment (female control *p* = 0.0039, female poly ICLC *p* = 0.0027; Figure 6M).

**Figure 7:**
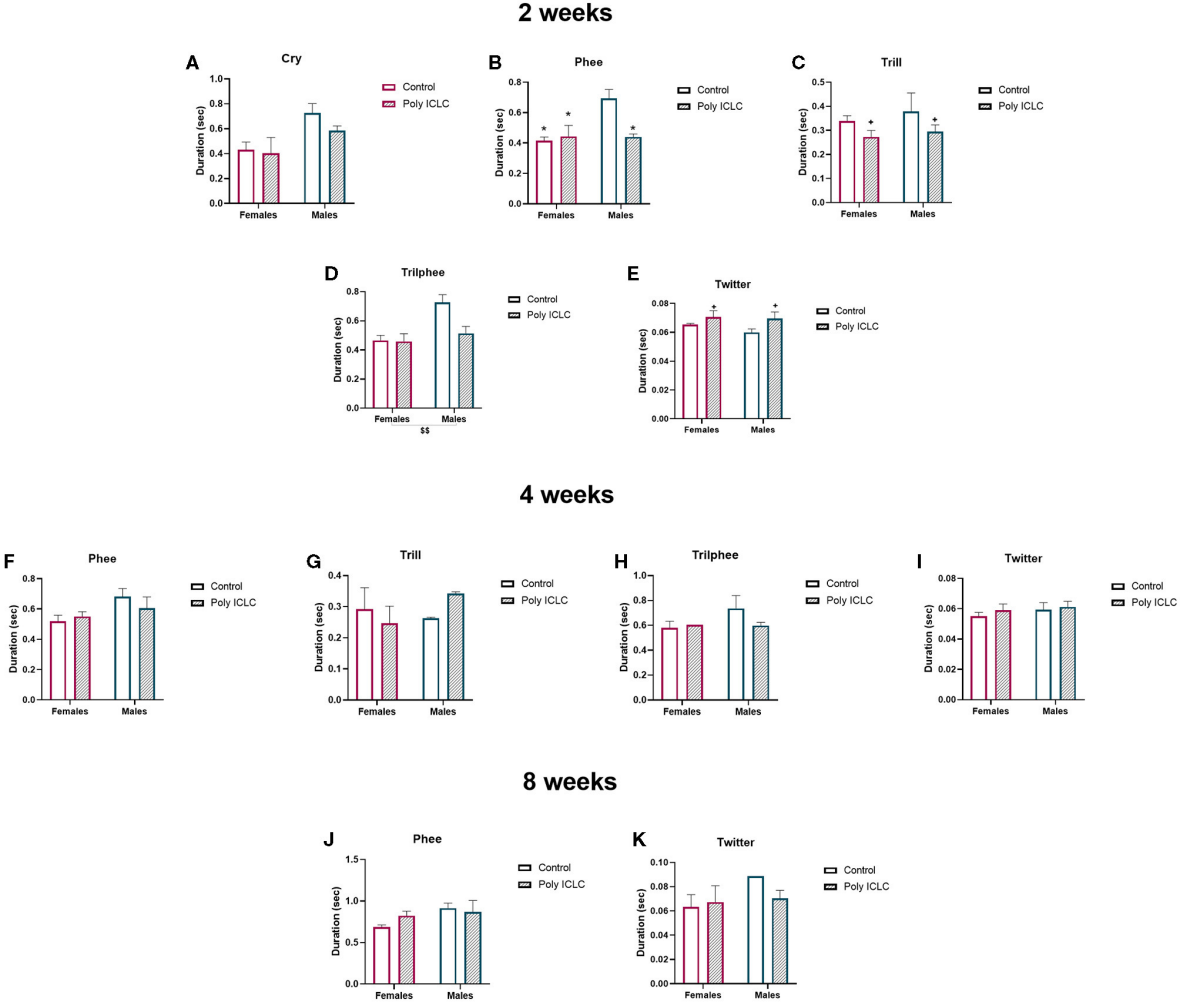
Duration of the different types of vocalizations is affected by MIA and sex at 2 weeks of age. Duration of each vocalization type at **(A–E)** 2 weeks of age, **(F–I)** 4 weeks of age, or **(J,K)** 8 weeks of age. Control female *n* = 6, Poly IC females *n* = 4, Control males *n* = 2, Poly IC males *n* = 3. Data are expressed as mean ± SEM and analyzed by Two-way ANOVA with Bonferroni *post-hoc*. Significant differences are indicated by *p < 0.05 vs. Male Control. Main effect of treatment +*p* < 0.05. Main effect of sex $$*p* < 0.01.

An additional vocalization parameter that has been shown to change in neurodevelopmental disruption models is the duration of each vocalization type (37). Thus, we calculated the average duration of all the vocalization types performed by the animals at each age. At 2 weeks of age, there were significant differences identified between sexes and treatments (Figures 7A–E). The duration of phees had an interaction between treatment and sex [*F*_(1,11)_ = 7.634, *p* = 0.0185], where the duration in control male was longer than poly ICLC males (*p* = 0.0465), control females (*p* = 0.0144), and poly ICLC female (*p* = 0.0369; Figure 7B). However, there was no effect of MIA in female marmosets (*p* = 0.964; Figures 7A–E). Duration of trill calls had a main effect of treatment, where both male and female MIA offspring presented shorter trills [*F*_(1,11)_ = 4.978, *p* = 0.0474; Figure 7C]. Trillphee duration was shorter in females when compared to males [sex effect, *F*_(1,11)_ = 9.813, *p* = 0.0095; Figure 7D]. Twitter duration increased in MIA offspring in both sexes [treatment effect, *F*_(1,11)_ =4.994, *p* = 0.0471; Figure 7E]. At 4 weeks of age, phee duration tended to be longer in males when compared to females [*F*_(1,11)_ = 4.119, *p* = 0.0673; Figure 7F]. However, at 8 weeks old, there were no statistically significant differences in call duration (Figures 7J,K).

**Figure 8:**
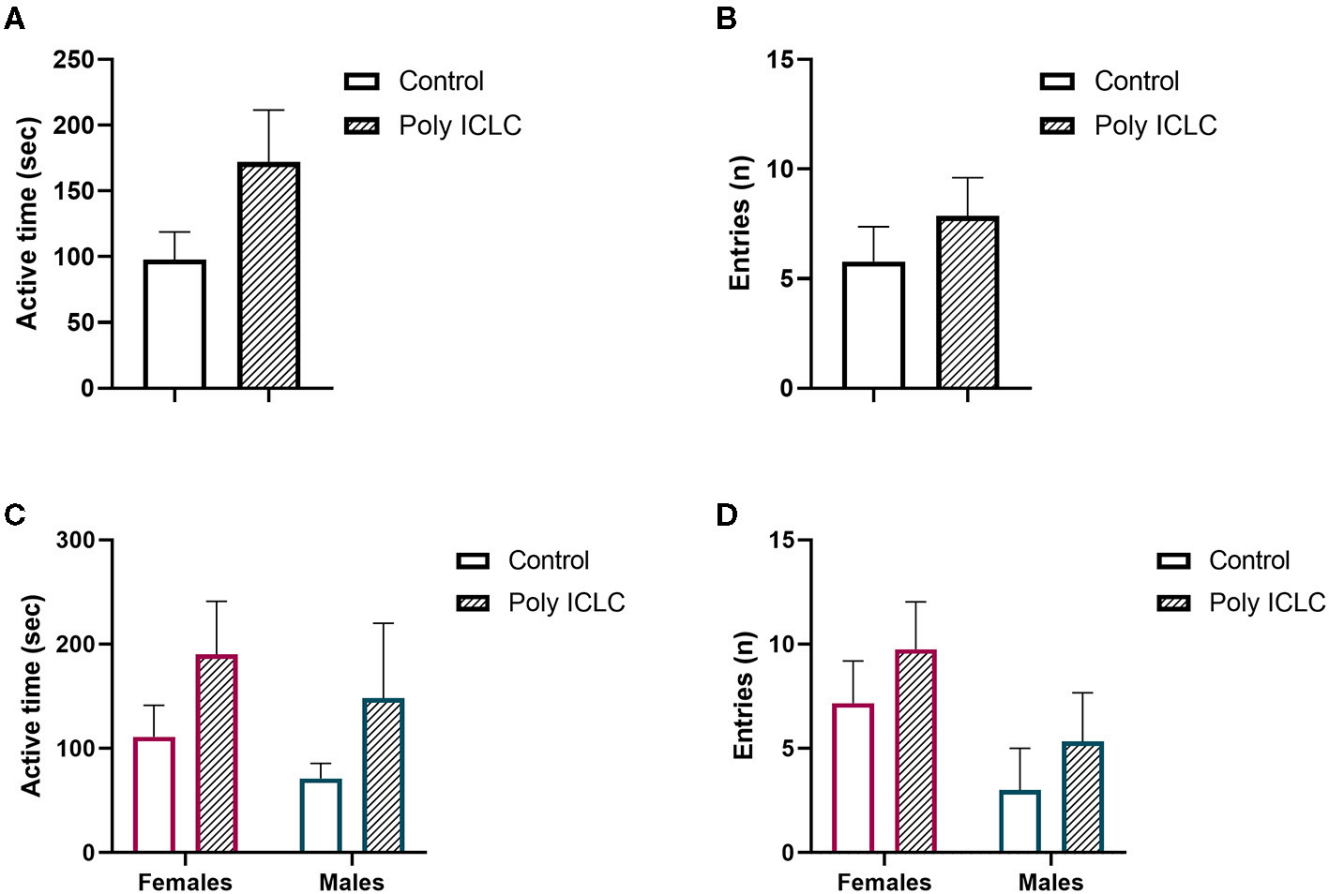
Offspring activity during habituation **(A,C)** total active time during 20 min of recordings. **(B,C)** Number of entries performed by offspring during recording. **(A,B)** Data pooled from females and males. **(C,D)** Data represented by sex. Control female *n* = 6, Poly IC females *n* = 4, Control males *n* = 3, Poly IC males *n* = 3. Data are expressed and mean ± SEM.

**Figure 9:**
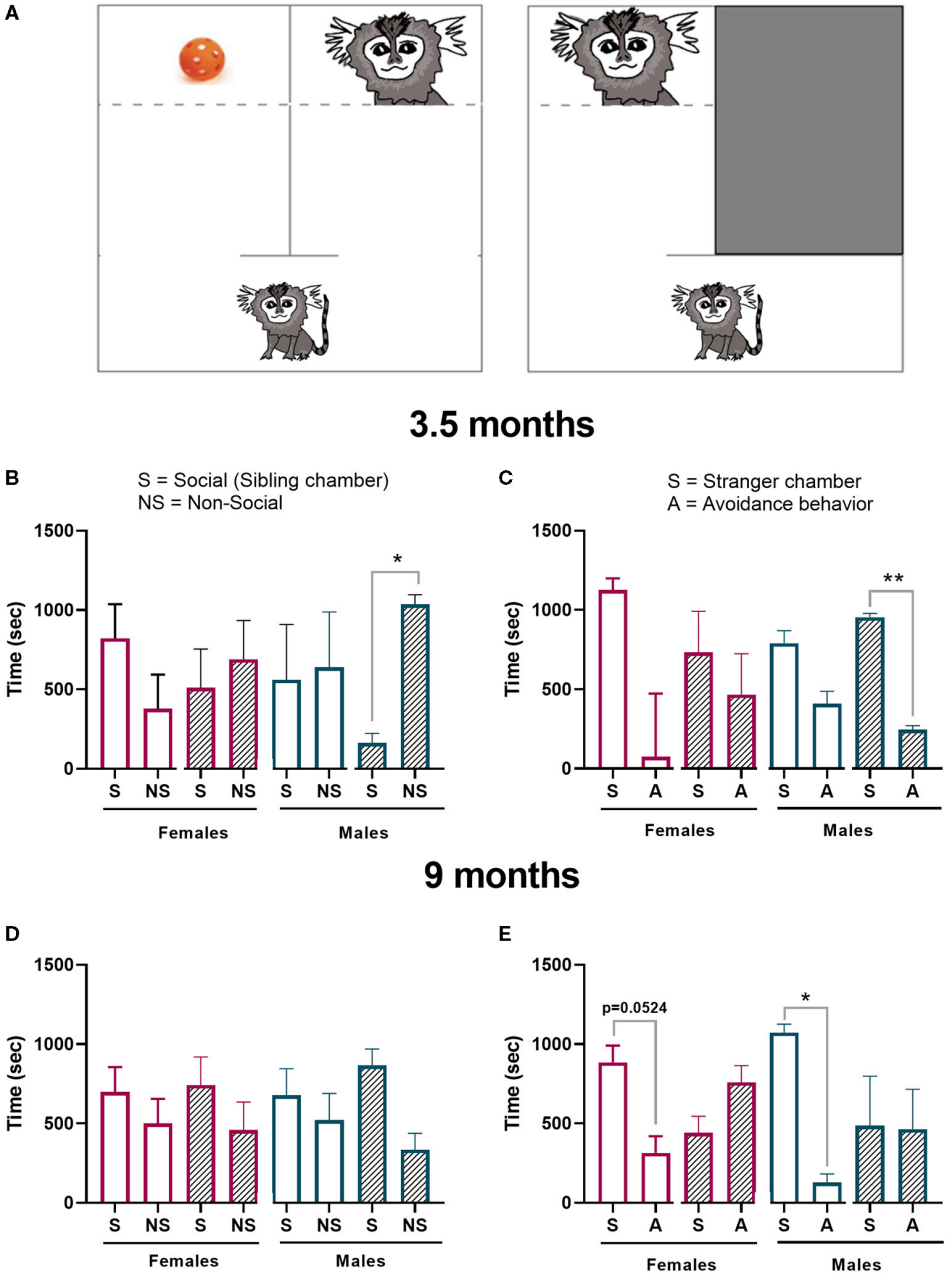
Sociability test. **(A)** Schematic of the test chamber. The first part of the test (social preference) the animal had the option to interact with a sibling (S) or a toy (NS). The second part of the test (social novelty) the animal had the option to interact with a stranger conspecific (S) or hide **(A)**. Offspring performance in the **(B)** sociability preference test or the **(C)** novelty preference test 3.5 months of age was measured. Offspring performance was again tested at 9 months in the **(D)** sociability preference test and **(E)** novelty preference test. Control female *n* = 6, Poly IC females *n* = 4, Control males *n* = 3, Poly IC males *n* = 3. Data are expressed as median± interquartile range and analyzed with Wilcoxon matched-pairs signed rank test. Normally distributed data are expressed as mean ± SEM and analyzed with paired *T*-test. Significant differences are indicated by *p < 0.05 and **p < 0.01.

A core feature of ASD and other neurodevelopment disorders is deficits in social behavior. In rodent studies, an established paradigm used to evaluate this core symptom is the three-chamber test. To assess if prenatal administration of poly ICLC to pregnant dams causes deficits in social behavior in marmoset offspring, we adapted the three-chamber test to be used for the marmosets. At the age of 3.5 months and 9 months, after 3 days of habituation, the animals were tested in the paradigm (Figures 8, 9). On the first day of habituation, for 20 min, 3.5-month-old marmoset activity was measured. Hyperactivity is a somewhat common characteristic of ASD (38, 39). While poly ICLC increased the mean time spent active and number of total chamber entries, the effect did not reach statistical significance between control and MIA offspring when both sexes were compared together (activity *p* = 0.0992; entries *p* = 0.3957) or separated (activity females *p* = 0.5219 and males *p* = 0.7064; entries females *p* = 0.8098 and males *p* = 0.9184; Figures 8A–D).

The infant's sociability test identified no significant differences between the time spent in the social and non-social chamber at 3.5 months of age in control females. However, male poly ICLC offspring spent a longer period in the non-social (toy) chamber when compared to the time it spent in the social chamber (t_2_, *p* = 0.0184; Figure 9B). In the stranger response test, both sexes in the control groups spent more time in the stranger chamber (S) than hiding (A). However, these differences did not reach significance. As opposed to the first sociability phase of the test, poly ICLC male offspring spent a significantly longer time in the stranger chamber than exhibiting avoidance behavior (t_2_, *p* = 0.0046; Figures 9B,C).

The sociability test during adolescence identified a preference to spent time in the sibling chamber independent of treatment. However, this preference did not reach statistical significance in any of the groups (Figures 9D,E). In the stranger response test, controls from both sexes showed a preference for spending time with the stranger over exhibiting avoidance behavior (females-t_4_, *p* = 0.0624, and males- t_2_, *p* = 0.0130; Figure 9E). In contrast, neither female nor male poly IC offspring exhibited any preference toward interacting with the stranger.

## Discussion

Here we establish and describe the characterization of a model of maternal immune activation in marmosets. Non-human primate models are an ideal translational tool as they present more complex behavior and are physiologically more similar to humans than rodents. Although there is considerable literature investigating the pathogenic mechanism of MIA in rodents, not much has been attempted to be replicated in primates. Bauman and collaborators have been establishing the rhesus monkey model of MIA during the last 6 years. Their research has identified not only deficits in behavior but also cellular alterations such as increased striatal dopamine levels and increased innate immune cytokines that are associated with behavioral deficits in MIA offspring (15, 17). While Rhesus Macaque non-human primate (NHP) models are the most widely utilized, they are hampered with several practical limitations not presented in marmosets, including longer gestational period, slow development reaching adulthood at 5 years of age, and difficulty to do studies evaluating interactions with other conspecific due to the animal's size and housing restrictions. Thus, developing a marmoset model to investigate the neurodevelopmental consequences of prenatal exposure to environmental ASD risk factors, such as MIA, provides several practical advantages. Marmosets, when compared to other non-human primates, are smaller in size, have a shorter gestation duration, faster development (reaching adulthood at 18 months of age), shorter lifespan (5–7 years), and the possibility of development of studies investigating their complex social interactions in the family unit.

To our knowledge, our data are the first to establish the poly ICLC MIA model in marmosets. Treatment with poly ICLC did not cause any significant overt developmental or physical disabilities as evidenced by equivalent Matscores across all treatment groups and sexes. Our study is modest in size making replication important. Nonetheless, preliminary evidence is encouraging, and the fact that we observed no differences in litter size when comparing poly ICLC group to saline-treated and non-treated controls, suggests no profound negative effect of poly ICLC in the pregnancy outcome (Table 2).

Offspring's behavior during their first 9 months of life, which roughly corresponds to the early adolescence (40), was assessed to identify if MIA offspring developed behavioral deficits. At birth, no motor deficits were identified in MIA offspring by analyzing their performance at the MATSCORE test (Figure 2A). During infancy, there were no differences in the marmoset's growth. However, by 37 weeks of age, when the animals are entering adolescence, female MIA-offspring were significantly heavier than controls. But the same effect was not seen in males (Figures 2D,E). Interestingly, new data emerging from the pediatric community and reported by the U.S. Centers for Disease Control indicate that adolescents with autism spectrum disorder are significantly more likely to be overweight or obese. This observation carries important implications for the risk of comorbidity and negative health outcomes in individuals with autism, but the underlying causes of this association remain speculative. Whether the increased body weight of female marmosets born to poly ICLC challenged dams reflects a translationally relevant feature of this preclinical model is an interesting possibility that warrants further investigation.

Communication deficits represent one of the core features of disorders such as ASD, and mouse models of MIA or genetic models of ASD (BTBR, CNTNAP2^−/−^, MECP2^−/−^) all exhibit a change in vocalization frequency when compared to controls (37, 38, 41). Thus, we assessed the offspring's vocal development by recording their vocalizations in isolation at 2, 4, and 8 weeks of age. Marmosets are known to present a diverse vocal repertoire that develops according to parental feedback (42). By 2 months of age, marmosets are expected to perform an adult-like repertoire (43). In our recordings, we identified five types of vocalization: cries, phees, trills, trillphees, and twitters (Figure 5A). Throughout the first 2 months of development, the total number of vocalizations performed by the infants decreased in both groups (Figure 3A). This behavior was expected as it has been shown to be common in this type of paradigm in marmosets (36). However, when separated by sex and age, we identified a decrease in the total number of vocalizations performed by MIA-offspring relative to same-sex controls in females (Figure 3D) but not in males (Figure 3G).

Additionally, because one of the dams in our colony provided both a control then a poly ICLC challenged litter, we were able to assess if different litters from the same dam could have similar vocal behavior independent of MIA, as genetics is also a susceptibility factor for neurodevelopmental disorders. Interestingly, when comparing two litters from the same dam, a control litter, and a poly ILCL litter, the pattern of vocalization development between the siblings of different litters was distinct. The control litter showed an inverted *U* shape curve, where it started with a lower number of vocalizations, increased by 4 weeks, and then decreased by 8 weeks. In contrast, poly ICLC litter began with a higher number of vocalizations that declined substantially as the infants got older (Figure 4). The interpretation of this data should be made with caution as the sample size was small, with only 2 infants per group. Additionally, the control group is represented by females while the treated group has one female and one male. Still, these results suggest that environmental factors contribute independently from genetics to impact the neurodevelopment of offspring. This notion is consistent with a study from Takahashi et al. which demonstrates that vocalization development is not fully genetic but also environmental (44).

Vocal learning is of great importance for most species. Vocalizations can be used for courtship, avoid predators, signalize food, and others (45). In marmosets, vocalization is as important as other species. Studies have shown that specific vocalizations can be used by marmosets to avoid contact with other species in the wild (46). Thus, learning the correct usage of vocalizations is imperative to the animal's safety in their natural habitat. Additionally, disturbances in the development of vocalization repertoire have been identified in several models of developmental disorders (47–49). Therefore, we analyzed the vocal repertoire of the animals in our study.

Analysis of vocal repertoire in our recordings identified five different types of calls: cries, twitters, trills, trillphees, and phees. The distribution of these calls changed throughout development and had differences between sexes. Most of the calls that were identified at 2 weeks of age disappeared by 8 weeks old (Figure 5B). The main calls identified by the 8 week recording are phees and twitters. Phee calls are used as long-distance calls to localize another conspecific. While twitters and trills are usually produced by adult marmosets during visual contact with a conspecific (36). In our paradigm, infants were isolated from any visual contact with the colony. But they were still able to hear the vocalizations performed by their conspecific. Thus, it is expected that with time they would learn what types of calls are used for long-distance and what calls are used when in visual contact. As the animals got older, they performed mainly the long-distance phee calls used when in indirect contact (non-visual) with the colony. Interestingly, at 8 weeks, marmosets born from poly ICLC challenged pregnancies tended to vocalize more twitters (visual contact calls) and fewer phees (long distance or agitation calls) than control offspring. This effect did not reach statistical significance as our pilot study was likely underpowered to detect more subtle, nuanced alterations in vocalization repertoire. In future studies, a more rigorous design targeted at testing this hypothesis should be employed.

Other calls, such as cries, are performed early in development when the marmoset infants still rely on their family to be fed and carried around (50). Marmosets are weaned around 3 months of age, so it is expected that at 2 months, they are learning which type of call is used in a given situation to survive independently. Interestingly, when isolating cry vocalizations by age, we identified that control males cry more than poly ICLC males and more than females independent of treatment. Also, male controls are the group that presents the high % of cry at 4 weeks of age (Figure 6). Together this data suggests that, at baseline, there is a dimorphic distribution of calls throughout development. Further, males may have been affected by MIA as they stop crying earlier when compared to controls, suggesting that isolation does not trigger the cry response upon separation from the family unit as it does in controls. This could potentially reflect a disruption in social connection. However, further studies with a larger number of animals are necessary to confirm this effect.

Duration of calls is another parameter that is analyzed in animal models of speech and vocal communication disorders. Our research identified the effects of MIA in the duration of calls in 2 weeks old marmosets. Trills and twitters duration were decreased and increased, respectively, in MIA offspring. Interestingly, for some vocalization types, there was a dimorphic effect, where male controls had a longer duration of phees and trillphee when compared to female offspring (Figure 7). At 4 and 8 weeks of age, there were no significant effects of sex or treatment in the main calls performed by the offspring in our paradigm.

Taken together, our communication data show that analysis of vocal repertoire demonstrates differences mainly at 2 weeks of age. A denser sampling frequency could be necessary to better assess differences in vocal development since marmosets rapidly develop, and we might have missed other alterations that occurred between our recordings. Additionally, as parental feedback is important for vocal development, future studies should evaluate the communication between infants and their colony in the MIA model. Especially for the cries-to-phee transition, which is contingent on parental response, not genetics, according to Takahashi et al. (44).

Deficits in sociability are common features of ASD and animal models of psychiatric and neurodevelopmental disorders. To evaluate social interactions in marmosets, we developed an adaptation of the three-chamber test that is routinely used in rodent studies (12, 51). Marmosets are highly exploratory of the social environment, but the nature of infant exploration in isolation to a new environment has not been extensively studied. In fact, to our knowledge, there are no studies in the literature that investigate specifically infant marmoset's social behavior in isolation and their response to stranger conspecifics. In our study, 3.5-month-old marmoset controls showed no preference between spending time in a chamber with an older sibling or in a chamber with a toy from their home cage. Male MIA-offspring did spend more time in the non-social chamber than in the social chamber, which replicated what is observed in rodent literature (12). However, female MIA offspring did not show the same behavior (Figure 9B). During the second part of the test, when exposed to a stranger conspecific, all control groups spent more time in the stranger chamber independently of treatment. Nonetheless, analysis of MIA-offspring identified that only MIA-males had a significant preference for the stranger chamber while female MIA-offspring had no preference. This data does not corroborate with rodent literature, where MIA-offspring commonly have no preference for social novelty (12, 51). This may reflect a more complex and nuanced social behavior in primates, and while anthropomorphic interpretation should be made with caution, MIA induced clear changes in the social behavior of male offspring relative to controls.

At 9 months of age, adolescent marmosets spent more time in the social chamber, but there were no significant differences between treatments and groups (Figure 9D). During the stranger response test, however, controls show a significant preference to spend time with the stranger, where MIA- offspring had no preference (Figure 9E). These data suggest adolescent marmosets perform similar to rodents in the stranger test during adolescence as control animals exhibit robust interest in novel conspecifics. Whereas, MIA offspring exhibit more social avoidance and no preference toward the stranger. It is not clear whether this reflects simply a lack of social interest in interacting with the stranger or an increase in social avoidance of the stranger.

The differences in behavior between infants and adolescent marmosets could be due to the ability of adolescents to better recognize stranger conspecifics as a new animal that they never had contact with before. Studies suggest that adult marmosets may recognize facial expressions of other marmosets (52), and the same might not be true for infants. Additionally, age could be an important factor corroborating to lack of significant social preference in our study since in rodents, where this test is traditionally used, the animals are typically sexually mature young adults when they are tested in the paradigm. Furthermore, measurements of direct interaction in rodents can be made by the test subject poking the recipient where the other animal is located. While measuring interaction in marmosets is more complex, the test subject can be touching the mash that separates it from the other conspecific while it is with their head turned to a different direction exploring the test chamber. Another point to be considered is that social interaction might just simply differ between infants and adolescents. For instance, at the age we tested the infant subjects, they were about to start to be weaned, which might imply that they are still depend on adults to be fed. While, at 9 months of age the animals are independent and that could possibly have influenced the results of our test.

For most primates, social bonds are crucial. The mains brain areas that constitute the social brain circuitry in humans are the orbitofrontal cortex, amygdala, temporal cortex, medial prefrontal cortex and anterior cingulate cortex (53). For non-human primates, similar brain regions have been shown to mediate social behavior. Old world monkeys' parietal and frontal cortex are activated in response to social interaction (54). Marmosets also show activation of frontal areas and temporal lobe in response to social interaction (55). Maternal immune activation in rodents can dysregulate social behavior which is associated to alteration in the frontal cortex. MIA disrupts the transcription of genes involved in the mTOR signaling cascade and glutamatergic neurotransmission in the frontal lobe (56). Studies have also shown that MIA alters the levels of cytokines throughout development in different brain areas including the frontal cortex. A study from Garay et al. (57) identified increased levels of IL-1α, IL-6, and IL-10 at postnatal day 60 in MIA offspring frontal cortex (57). Other cytokines were decreased in the frontal and cingulate cortex at postnatal day 14, such as IFN-y. The role of IFN-y in social behavior have been previously described by Filiano et al. (58), where they demonstrated IFN-y can elevate tonic GABAergic inhibition and regulate social behavior as IFN-y knockout mice present lack of social preference in the three chamber test (58). It is out of the scope of our study to identify a mechanism by which MIA disrupted social behavior in marmosets since we have not collected data from the subject's brains. However, we can hypothesize that MIA in marmosets present similar mechanisms that have been previously identified in rodent studies. And those mechanisms might be responsible for the effects of prenatal inflammation in the behavioral outcomes we measured. Further studies are necessary to characterize the effect of MIA in the development of marmoset's brains and if the consequences of prenatal inflammation in primates are similar to what is seen in rodents.

Taken together, we show that MIA offspring present communication and sociability deficits during their first 9 months of life. While we had been able to establish and begin characterizing the effect of prenatal administration of poly ICLC in marmoset's offspring development, more studies are necessary to fully characterize the consequences of MIA in this species' development. Specifically, more frequent sampling and more sensitive tests could better characterize changes in social behavior. Further, the addition of cognitive testing that was outside the scope of our pilot project would be an important expansion of the research. Also, it is important to point out that the age when we evaluated vocal development in the marmosets is correspondent to 2 years of age in humans, which is the period when children start to be diagnosed with autism. While assessment of social behavior in our study was done in adolescent marmosets. Thus, further characterization of the animal's phenotype until adulthood is necessary to identify what other types of deficits they might develop with age.

## Data Availability Statement

The raw data supporting the conclusions of this article will be made available by the authors, without undue reservation.

## Ethics Statement

The animal study was reviewed and approved by IACUC committee at Texas Biomedical Research Institute.

## Author Contributions

DSC carried out experiments, collected and analyzed the data, and prepared the initial manuscript draft and incorporated subsequent edits from DL-C, RV, CR, ST, and JCO. DL-C managed the marmoset colony, performed all Matscore evaluations, and supported experiments. RV analyzed and summarized vocalization data and supported experiments. CR and ST served as project sponsors at Southwest National Primate Center, assisted with experimental design, colony management, and manuscript editing. JCO conceived the pilot study, coordinated the research team, oversaw data collection and analysis, and prepared the final manuscript. All authors contributed to the article and approved the submitted version.

## Conflict of Interest

The authors declare that the research was conducted in the absence of any commercial or financial relationships that could be construed as a potential conflict of interest.

## Publisher's Note

All claims expressed in this article are solely those of the authors and do not necessarily represent those of their affiliated organizations, or those of the publisher, the editors and the reviewers. Any product that may be evaluated in this article, or claim that may be made by its manufacturer, is not guaranteed or endorsed by the publisher.

## Supplementary Material

Supplementary Figure 1 Induction of TNF-α in response to Poly ICLC. Pregnant marmosets were treated with saline (*n* = 3) or 5 mg/kg of Poly ICLC (*n* = 8) at gestational day 63, 65, and 67. Blood collection was done at baseline and 2 h after the first and third injection. Data is represented as percentage of baseline levels of TNF-α and analyzed with One-Way ANOVA. Significant differences are indicated by **p* < 0.05.

Supplementary Table 1 Description of the pregnancy of dams used in the study. Number of previous pregnancies and miscarriages. Size of the litter used in the study during gestation. Total number of offspring that survived after birth. Sex of the offspring that survived.

Supplementary Table 2 Detailed data from the MASTCORE task. Data is presented as mean ± SEM. Data were analyzed using One-way ANOVA.
