# Supplementary figures and images for: Advancing Autism Research From Mice to Marmosets: Behavioral Development of Offspring Following Prenatal Maternal Immune Activation
Source: Front Psychiatry. 2021 Aug 6;12:705554. doi: 10.3389/fpsyt.2021.705554 (PMC8377364; doi:10.3389/fpsyt.2021.705554)

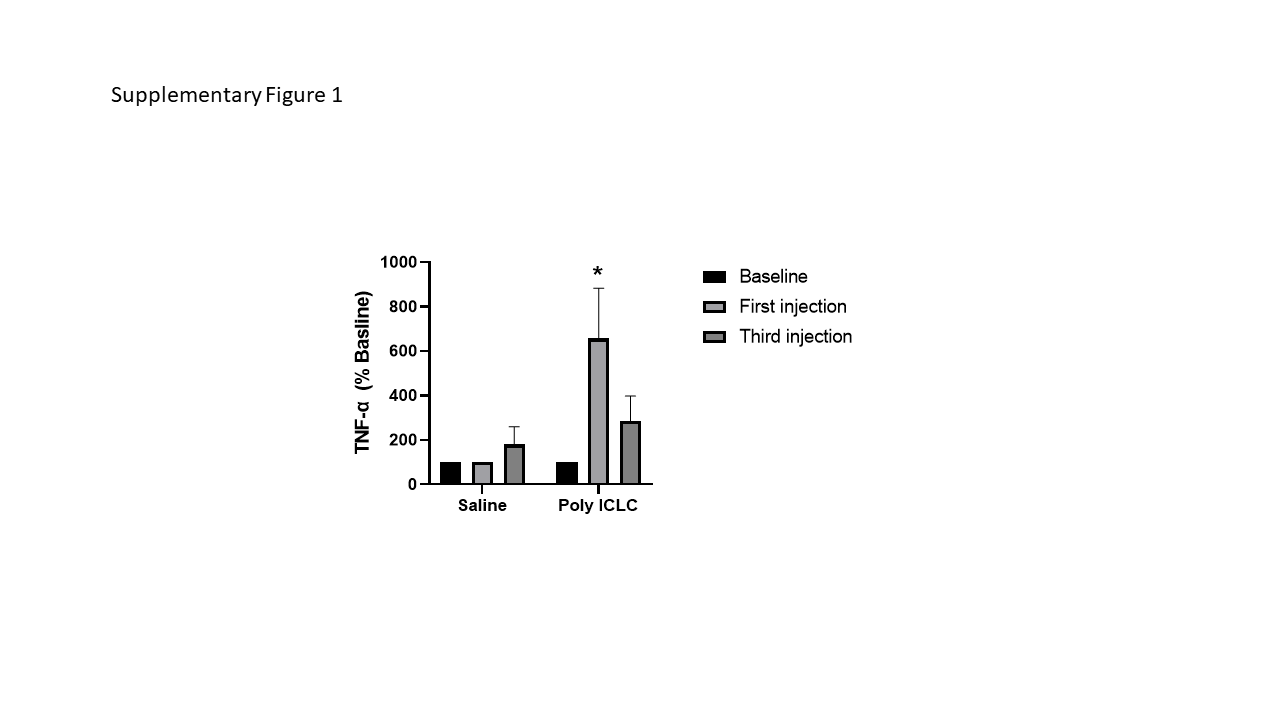

Supplement: Supplementary Figure 1 — Induction of TNF-α in response to Poly ICLC. Pregnant marmosets were treated with saline (n = 3) or 5 mg/kg of Poly ICLC (n = 8) at gestational day 63, 65, and 67. Blood collection was done at baseline and 2 h after the first and third injection. Data is represented as percentage of baseline levels of TNF-α and analyzed with One-Way ANOVA. Significant differences are indicated by *p < 0.05. [file Image_1.TIF]
