# Supplementary material for: Advancing Autism Research From Mice to Marmosets: Behavioral Development of Offspring Following Prenatal Maternal Immune Activation
Source: Front Psychiatry. 2021 Aug 6;12:705554. doi: 10.3389/fpsyt.2021.705554 (PMC8377364; doi:10.3389/fpsyt.2021.705554)
Supplement: Supplementary Table 1 — Description of the pregnancy of dams used in the study. Number of previous pregnancies and miscarriages. Size of the litter used in the study during gestation. Total number of offspring that survived after birth. Sex of the offspring that survived. [file Table_1.docx]

| Dam ID | Prenatal treatment | Previous pregnancies | Previous miscarriages | Litter size | Delivered? | Total offspring survived the first month | Offspring ID |
| --- | --- | --- | --- | --- | --- | --- | --- |
|  |  |  |  |  |  |  | (sex) |
| *32873* | No treatment | 4 | 0 | 4 | yes | 2 | 37293 (F) 37295 (M) |
| *32873* | No treatment | 4 | 0 | 3 | yes | 2 | 37637 (M) |
| *33586* | No treatment | 3 | 0 | 3 | yes | 2 | 37371 (F) 37372 (F) |
| *32549* | No treatment | 5 | 0 | 3 | yes | 2 | 38144 (F) 38145 (F) |
| *32873* | MIA | 4 | 0 | 2 | yes | 0 | - |
| *32549* | MIA | 5 | 0 | 3 | yes | 2 | 37866 (M) 37867 (F) |
| *34732* | MIA | 1 | 0 | 1 | yes | 1 | 38441 (F) |
| *36581* | MIA | 1 | 0 | 2 | No | - | - |
| *35070* | MIA | 1 | 0 | 4 | yes | - | - |
| *35070* | MIA | 2 | 1 | 4 | yes | 2 | 38302 (F) 38303 (F) |
| *35071* | MIA | 3 | 0 | 2 | yes | 1 | 38183 (F) |
| *36693* | MIA |  |  | 3 | yes | 2 | 38637 (M) 38368 (M) |
| *36818* | Saline | 1 | 0 | 3 | yes | 1 | 39059 (F) |
| *36266* | Saline | 4 | 0 | 0 | no | - | - |
| *36054* | Saline | 2 | 0 | 3 | yes | 3 | 39208 (M) 39209 (F) 39210 (M) |

**Supplementary table 1. Description of the pregnancy of dams used in the study.** Number of previous pregnancies and miscarriages. Size of the litter used in the study during gestation. Total number of offspring that survived after birth. Sex of the offspring that survived.
