# Supplementary material for: Advancing Autism Research From Mice to Marmosets: Behavioral Development of Offspring Following Prenatal Maternal Immune Activation
Source: Front Psychiatry. 2021 Aug 6;12:705554. doi: 10.3389/fpsyt.2021.705554 (PMC8377364; doi:10.3389/fpsyt.2021.705554)
Supplement: Supplementary Table 2 — Detailed data from the MASTCORE task. Data is presented as mean ± SEM. Data were analyzed using One-way ANOVA. [file Table_2.docx]

| Group | SEX | Number of infants | MATSCORE tasks data | | | | | | | | | | | | | MATSCORE |
| --- | --- | --- | --- | --- | --- | --- | --- | --- | --- | --- | --- | --- | --- | --- | --- | --- |
|  |  |  | Crawling | Clasping | Righting 1 | Latency to right 1 (sec) | Clasping 2 | Righting 2 | Latency to right 2 | Orient up score | Latency to turn head up | Rooting (Left) | Rooting (Right) | Auditory orientation (Left) | Auditory orientation (Right) |  |
| Non-treated controls | F | 3 | 1 ± 0 | 1 ± 0 | 2 ± 0 | 23.33 ± 17.55 | 1 ± 0 | 2± 0 | 23.3 | 1.67 ± 0.58 | 32.67 ± 23.44 | 1.33 ± 0.58 | 1.67 ± 0.58 | 0.67 ± 1.15 | 0.66 ± 1.15 | 12.68± 3.79 |
|  | M | 2 | 1 ± 0 | 1 ± 0 | 1 ± 1.41 | 34 ± 36.76 | 1.5 ± 0.71 | 2± 0 | 12.50 ± 0.71 | 2 ± 0 | 20.95 ± 18.46 | 1 ±1.41 | 1.5 ± 0.71 | 1 ± 1.41 | 0.5 ± 0.7 | 12.50 ± 3.54 |
| Poly ICLC treated | F | 4 | 1.16 ± 0.76 | 1.33 ± 0.57 | 2 ± 0 | 35 ± 2.64 | 1.5 ± 0.57 | 2 ± 0 | 29.50 ± 24.19 | 2 ± 0 | 14.03 ± 12.66 | 1 ± 0.82 | 1.5 ± 1 | 0.63 ± 0.95 | 0 ± 0 | 12.75 ± 2.72 |
|  | M | 3 | 1 ± 0 | 2 ± 0 | 2 ± 0 | 32 ± 8.48 | 1.33 ± 0.58 | 2± 0 | 20.67 ± 4.04 | 2 ± 0 | 10 ± 1 | 1.33 ± 0.58 | 0.67 ± 0.58 | 0.83 ± 0.29 | 1.33 ± 1.15 | 13.83± 2.02 |
| Saline treated Controls | F | 3 | 0.66 ± 0.44 | 1.33 ± 0.57 | 2 ± 0 | 18.75 ± 7.93 | 1.33 ± 0.58 | 2± 0 | 23 ± 5.29 | 1.33 ± 0.58 | 36 ± 30.27 | 1 ± 0 | 0.5 ± 0.71 | 0 ± 0 | 0 ± 0 | 11 ± 2.95 |
|  | M | 2 | 1 ± 0 | 2 ± 0 | 2 ± 0 | 32 ± 8.48 | 2 ± 0 | 2±0 | 18 ± 5.66 | 2 ± 0 | 5.5 ± 0.71 | 1 ± 0 | 2 ± 1.41 | 2 ± 0 | 1 ± 1.41 | 15 ± 2.82 |

**Supplementary Table 2. Detailed data from the MASTCORE task. Data is presented as** mean ± SEM. Data were analyzed using One-way ANOVA.
